# Interstitial lung disease induced by docetaxel and ramucirumab chemotherapy after nivolumab treatment

**DOI:** 10.1002/rcr2.564

**Published:** 2020-04-22

**Authors:** Komugi Okeya, Yukio Kawagishi, Mako Yamoto, Mami Shimizu, Toshihide Imizuda, Hiroshi Tsuji

**Affiliations:** ^1^ Department of Respiratory Medicine Kurobe City Hospital Kurobe Japan

**Keywords:** Docetaxel, immune checkpoint inhibitor, interstitial lung disease, nivolumab, ramucirumab

## Abstract

Three men (aged 64, 65, and 67 years) with advanced lung cancer who had been treated with nivolumab developed interstitial lung disease (ILD) during chemotherapy with docetaxel and ramucirumab. The treatment was clinically effective; however, the patients experienced immune‐related adverse effects due to nivolumab therapy: two patients developed ILD and the third developed psoriasis. Because the patients showed progression, docetaxel and ramucirumab chemotherapy was administered. Although two patients showed a clinical response, all patients developed grade 3 ILD during therapy. Furthermore, the patients developed respiratory failure and needed corticosteroid therapy. Although their condition improved owing to the therapy, the patients could not receive additional cancer treatment and died of cancer. On the basis of the results obtained, we speculated that although the regimen of docetaxel and ramucirumab after nivolumab therapy might be effective against non‐small cell lung cancer, it might increase the risk for ILD in some patients.

## Graphical abstract

We report cases of three patients who developed interstitial lung disease (ILD) during combination therapy with docetaxel and ramucirumab after nivolumab treatment.

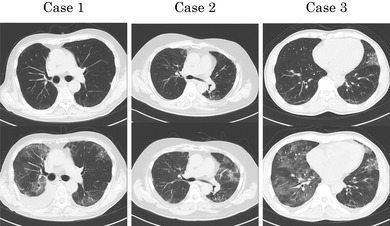

## Introduction

Nivolumab (an anti‐programmed death ligand 1 (PD‐L1) antibody) is the first approved immune checkpoint inhibitor (ICI) for the treatment of non‐small cell lung cancer (NSCLC) in Japan. It has unique clinical efficacy, different from that of conventional chemotherapies, and has been widely used for previously treated NSCLC in clinical practice. However, immune‐related adverse effects (irAEs) such as interstitial lung disease (ILD) or diabetes mellitus type I are known to develop in nivolumab‐treated patients with NSCLC 1. On the other hand, the combination of docetaxel and ramucirumab as a second‐line treatment in patients with stage IV NSCLC has been reported to improve the median survival time compared to that associated with docetaxel therapy alone (REVEL trial) 2. Herein, we report cases of three patients who developed ILD during combination therapy with docetaxel and ramucirumab after nivolumab treatment.

## Case Series

### Case 1

**Figure 1:**
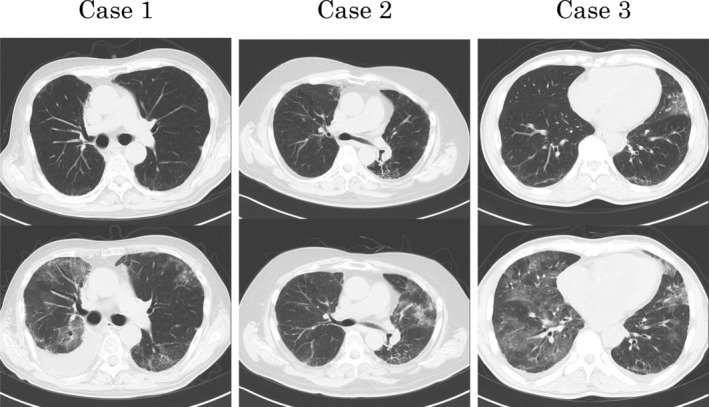
Thoracic computed tomography of the three patients. Compared with the images of the upper row obtained before the onset of interstitial lung disease (ILD), images of the lower row show diffuse ground‐glass opacities in all cases at the onset of ILD.

A 59‐year‐old male ex‐smoker was diagnosed with combined small cell carcinoma and adenocarcinoma (pT1bN2M0 stage IIIA) based on surgical biopsy. He had limited small cell carcinoma, for which concurrent chemoradiotherapy with cisplatin and etoposide was performed along with accelerated hyperfractionated radiotherapy (30 Gy) followed by prophylactic cranial irradiation (30 Gy). Two years after the diagnosis, however, recurrence was noted. Thereafter, the patient sequentially underwent three regimens of chemotherapy with amrubicin, carboplatin and irinotecan, and topotecan. Approximately 3.5 years after the diagnosis, nivolumab was administered as the fifth‐line chemotherapy due to progressive disease (PD). The therapy resulted in partial response (PR); however, the patient developed psoriasis. After eight months of the therapy, the disease progressed. Computed tomography (CT) performed at this point showed very mild patchy opacities scattered in the peripheral lung, which were thought to not hinder therapy. Chemotherapy with docetaxel and ramucirumab was started as the sixth‐line treatment, and pegfilgrastim was used to prevent febrile neutropenia. Although the chemotherapy resulted in stable disease (SD), on day 18 of the third course, the patient visited our hospital due to fever and dyspnoea. He also had hypoxaemia, and chest CT revealed diffuse ground‐glass opacities (GGOs) in the lung fields (Fig. 1). On the basis of the findings, a diagnosis of grade 3 ILD associated with chemotherapy was made. Accordingly, 40 mg prednisolone was administered; ILD showed improvement over several days. However, mild fibrosis and infiltration persisted five months after the onset of ILD, and the patient could not receive any chemotherapy due to the risk of ILD exacerbation and died of cancer 12 months after ILD onset.

### Case 2

A 65‐year‐old male smoker with left shoulder pain was diagnosed with locally advanced squamous cell carcinoma of the lung (cT3N1M0 stage IIIA) and received concurrent chemoradiotherapy with cisplatin and S‐1 and 60 Gy of thoracic radiation, which resulted in PR and pain alleviation. One year later, the patient again developed left shoulder pain, and CT revealed PD. Therefore, nivolumab was started as second‐line chemotherapy. Thereafter, the pain alleviated, and the tumour shrunk by six weeks after nivolumab treatment. CT performed at 12 weeks after nivolumab treatment revealed bilateral diffuse GGO in the lungs. Although the patient did not show respiratory symptoms, 20 mg prednisolone was administered for grade 2 ILD due to nivolumab. The pulmonary opacities improved over two weeks, after which prednisolone was tapered and nivolumab treatment was restarted. Eight months after the first administration of nivolumab, disease progression was noted. CT showed that a few mild patchy opacities persisted in the peripheral lungs, which were thought not to hinder therapy. Therefore, docetaxel and ramucirumab were started as third‐line chemotherapy; pegfilgrastim was also used. Although the chemotherapy resulted in SD, the patient visited our hospital with a complaint of fatigue on day 5 of the third course. He had developed respiratory failure due to grade 3 ILD. Chest CT showed diffuse pulmonary infiltration (Fig. 1). Respiratory failure improved after starting 30 mg prednisolone; however, a considerable pulmonary lesion remained. Seven months thereafter, the patient died of cancer without any additional chemotherapy.

### Case 3

The patient was a 65‐old‐year male ex‐smoker who visited our hospital with a complaint of hoarseness. He was diagnosed with metastatic lung adenocarcinoma (cT1cN3M1 stage IV) without any driver mutation (epidermal growth factor receptor gene mutation, echinoderm‐microtubule‐associated protein‐like 4‐anaplastic lymphoma kinase (EML4‐ALK) fusion protein, or c‐ros oncogene 1 (ROS1) fusion protein) and with weak PD‐L1 expression. Four cycles of carboplatin and pemetrexed were administered as the first regimen followed by three cycles of pemetrexed as maintenance therapy. CT performed thereafter showed PD, because of which nivolumab was started. CT performed subsequently showed PR; however, pulmonary infiltration was noted in the left upper lung lobe. Although we suspected the development of grade 1 ILD, therapy was continued because of a mild lesion and no symptom. Six months after nivolumab therapy, the disease progressed. Thus, docetaxel and ramucirumab were started as the third‐line regimen; pegfilgrastim was also used. Three weeks later, the patient visited the hospital due to fatigue. He presented with respiratory failure and had developed grade 3 ILD (Fig. 1). Therefore, 40 mg prednisolone was started, after which respiratory failure and ILD improved. He died of cancer five months after the onset of ILD.

## Discussion

We reported cases of three patients with lung cancer who developed ILD during chemotherapy with docetaxel and ramucirumab after nivolumab treatment. Nivolumab therapy was clinically effective in these patients; however, they developed irAEs (two patients developed ILD and the third developed psoriasis) (Table 1). The development of irAEs has been reported to be related to preferred outcomes 3, 4. Although the mechanisms underlying the association are unknown, nivolumab has been thought to successfully activate the host immune system. On the other hand, chemotherapy with docetaxel and ramucirumab after ICI treatment was reported to show high efficacy compared to that noted in cases without the previous use of ICI treatment 5, 6. It has been suggested that the high efficacy of docetaxel and ramucirumab after ICI treatment is attributable to the following two mechanisms. The first mechanism is alteration of the tumour and/or microenvironment by ICI treatment resulting in increased sensitivity to cytotoxic agents. The other mechanism is the combination of cytotoxic and antiangiogenic agents resulting in an intensified ICI treatment effect 6. However, Harada et al. reported that docetaxel and ramucirumab chemotherapy after ICI treatment induced ILD in three out of 18 patients 6. Although CT findings showed similar diffuse GGO in our three patients, we could not obtain pathological specimens and the aetiology remains unclear. Previous ICI treatment may also enhance the risk for ILD due to chemotherapy. Conversely, chemotherapy may trigger ILD as a late‐onset irAE. Docetaxel and ramucirumab chemotherapy was reported to induce ILD (10.5%) in a Japanese phase II trial, although the docetaxel dose was lower (60 mg/m^2^) in the Japanese regimen than in the REVEL trial (75 mg/m^2^) 2, 7. Drug‐induced ILD was reported to frequently develop in Japanese patients compared to that in patients from other countries 8. A series of treatments including radiotherapy is associated with various types of damage and immune modification among patients. We speculate that therapy with docetaxel and ramucirumab after nivolumab treatment could carry a high risk of ILD, although sequentially administered regimens could be highly efficacious against NSCLC.

**Table 1:** Characteristics and clinical courses of the patients.

	Case 1	Case 2	Case 3
Age (years)	64	67	65
Sex	Male	Male	Male
Smoking history	Ex‐smoker (15 pack‐year)	Smoker (30 pack‐year)	Ex‐smoker (40 pack‐year)
Histology	Small cell carcinoma combined with adenocarcinoma	Squamous cell carcinoma	Adenocarcinoma
PD‐L1/22C3 (TPS)	<1%	No data	40–50%
No. of prior regimens before Nivo therapy	4	1	1
Thoracic radiotherapy	30 Gy	60 Gy	None
No. of Nivo administration	13	14	13
Response to Nivo (maximum reduction)	PR (−43%)	PR (−40%)	PR (−30%)
irAE	Psoriasis	ILD (grade 2)	ILD (grade 1)
No. of DOC/RAM administration	3	3	1
Response to DOC/RAM (maximum reduction)	SD (+5%)	SD (−27%)	NE
Grade of ILD due to DOC/RAM	Grade 3	Grade 3	Grade 3
PSL dose for ILD (mg)	40	30	40
Outcome of ILD	Improvement	Improvement	Improvement
Survival after ILD onset (months)	12	6	5

DOC, docetaxel; ILD, interstitial lung disease; irAE, immune‐related adverse effect; NE, not evaluable; Nivo, nivolumab; PD‐L1, programmed death ligand 1; PR, partial response; PSL, prednisolone; RAM, ramucirumab; SD, stable disease; TPS, tumour proportion score.

Our patients may have been particularly vulnerable due to a history of nivolumab‐induced ILD or thoracic irradiation (Table 1). Our experience, however, was limited to a small number of cases in which docetaxel and ramucirumab chemotherapy was used after nivolumab treatment. Although further data are needed to evaluate the risk of ILD induced by chemotherapy after nivolumab, the risk of ILD development should be considered while planning the therapeutic strategy for NSCLC, which could be complicated and prolonged.

### Disclosure Statement

Appropriate written informed consent was obtained for publication of this case series and accompanying images.

This study was approved by ethics committee of Kurobe City Hospital (approval number: c003‐2020).
